# Target Therapies for Uterine Carcinosarcomas: Current Evidence and Future Perspectives

**DOI:** 10.3390/ijms18051100

**Published:** 2017-05-20

**Authors:** Salvatore Giovanni Vitale, Antonio Simone Laganà, Stella Capriglione, Roberto Angioli, Valentina Lucia La Rosa, Salvatore Lopez, Gaetano Valenti, Fabrizio Sapia, Giuseppe Sarpietro, Salvatore Butticè, Carmelo Tuscano, Daniele Fanale, Alessandro Tropea, Diego Rossetti

**Affiliations:** 1 Unit of Gynecology and Obstetrics, Department of Human Pathology in Adulthood and Childhood “Gaetano Barresi”, University of Messina, 98125 Messina, Italy; vitalesalvatore@hotmail.com (S.G.V.); antlagana@unime.it (A.S.L.); 2 Department of Obstetrics and Gynecology, Campus Bio Medico University of Rome, 00128 Rome, Italy; s.capriglione@unicampus.it (S.C.); angie_cest@hotmail.com (R.A.); s.lopez@unicampus.it (S.L.); 3 Unit of Psychodiagnostics and Clinical Psychology, University of Catania, 95124 Catania, Italy; 4 Department of General Surgery and Medical Surgical Specialties, University of Catania, 95124 Catania, Italy; valentigaetano@gmail.com (G.V.); fabrizio.sapia@libero.it (F.S.); sarpietrogiuseppe@gmail.com (G.S.); alessandrotropea@aol.com (A.T.); 5 Department of Human Pathology, Unit of Urology, University of Messina, 98124 Messina, Italy; salvobu@gmail.com; 6 Radiation Oncology Department, AO “Bianchi-Melacrino-Morelli”, 89124 Reggio Calabria, Italy; carmelotuscano@hotmail.it; 7 Department of Surgical, Oncological and Oral Sciences, Section of Medical Oncology, University of Palermo, 90127 Palermo, Italy; fandan@libero.it; 8 Unit of Gynecology and Obstetrics, Desenzano del Garda Hospital, Section of Gavardo, 25085 Gavardo, Brescia, Italy; docrossetti@gmail.com

**Keywords:** carcinosarcomas, uterine cancer, genetics, epigenetics, immunotherapy

## Abstract

Carcinosarcomas (CS) in gynecology are very infrequent and represent only 2–5% of uterine cancers. Despite surgical cytoreduction and subsequent chemotherapy being the primary treatment for uterine CS, the overall five-year survival rate is 30 ± 9% and recurrence is extremely common (50–80%). Due to the poor prognosis of CS, new strategies have been developed in the last few decades, targeting known dysfunctional molecular pathways for immunotherapy. In this paper, we aimed to gather the available evidence on the latest therapies for the treatment of CS. We performed a systematic review using the terms “uterine carcinosarcoma”, “uterine Malignant Mixed Müllerian Tumors”, “target therapies”, “angiogenesis therapy”, “cancer stem cell therapy”, “prognostic biomarker”, and “novel antibody-drug”. Based on our results, the differential expression and accessibility of epithelial cell adhesion molecule-1 on metastatic/chemotherapy-resistant CS cells in comparison to normal tissues and Human Epidermal Growth Factor Receptor 2 (HER2) open up new possibilities in the field of target therapy. Nevertheless, future investigations are needed to clarify the impact of these new therapies on survival rate and medium-/long-term outcomes.

## 1. Introduction

Carsinosarcoma (CS), also defined as Malignant Mixed Müllerian Tumor (MMMT), is one of the less common and most challenging tumors to treat. It consists of a mixture of carcinomatous (malignant epithelial) and sarcomatous (mesenchymal) components. The first component is commonly serous, although it can be endometrioid, clear cell, squamous cell or undifferentiated carcinoma; the second component can be homologous (tissue native to the primary site) or heterologous [1,2]. CS can arise from each organ of the female reproductive tract, including the endometrium, ovary, cervix, vulva or fallopian tube. Nevertheless, the most frequent primary site is the endometrium, with late differentiation into the sarcomatous components. Both components express very similar immunohistochemical markers, share common somatic mutations, and often show identical X chromosome inactivation patterns [3]. Immunohistochemistry allows us to define the individual components of the specific histotype; i.e., the rhabdomyoblastic component should express MyoD1, desmin, or myogenin, while the serous component should express p53 and cytokeratins such as epithelial membrane antigen (EMA). In any case, it is established that the sarcomatous component can express cytokeratins and the epithelial element is often immunoreactive for vimentin. These results reflect the common mesodermal origin of these two tumors. Endometrial stromal tumors could be diagnosed using the Cluster of Differentiation 10 (CD10) marker, which is expressed in its homologous component. In the presence of experienced and dedicated pathologists, immunohistochemistry is not mandatory for a proper diagnosis, but may be useful in solving doubts in specific cases such as confirming the presence of rhabdomyoblasts in the specimen. In general, uterine sarcoma represents 3–5% of all corpus uteri malignancies, occurring in 1–4 per 1000 women in the USA [4]. CS shares similar risk factors with endometrial carcinomas; both of them are associated with obesity, nulliparity, and the use of exogenous estrogen and tamoxifen [5,6,7]. Conversely, progestin-containing contraceptives are protective against both types of neoplasms. A history of exposure to pelvic radiation is also associated with an increased risk of developing uterine carcinosarcoma [8,9]. Women affected by CS may present pain, bleeding, and rapid growth of the uterine volume [10]. Over 10% of patients affected by CS will have metastatic disease, and 60% will have extrauterine recurrence [11]. In the case of CS limited to the pelvic cavity and abdomen, surgery should be considered as a primary intervention in order to allow both staging and initial treatment [12]. According to the International Federation of Gynecology and Obstetrics (FIGO) guidelines, it is mandatory to perform an accurate surgical staging, including total hysterectomy, bilateral salpingo-oophorectomy, pelvic and para-aortic lymph node dissection, peritoneal cytology, omentectomy, and peritoneal biopsies.

In many cases, lymphatic and blood system invasion happens. Metastatic and recurrent tumors may be exclusively carcinomatous, sarcomatous, or mixed, but they are predominantly carcinomatous. Some particular histotypes such as serous and clear cell carcinomas are associated with negative prognostic factors such as local and distant metastases, cervical involvement, deep myometrial invasion and lymphovascular space invasion. Furthermore, the presence of heterologous elements is correlated with poor prognostic factor in stage I patients. The role of adjuvant therapy (AT) (radiotherapy, chemotherapy or chemoradiation-therapy) remains uncertain. There seems to be a slight improvement in disease-free survival rate and local control for patients subjected to radiotherapy as AT [13]. Although available data so far are not so robust as to draw firm conclusions, it is reasonable to try to perform a complete cytoreduction (no gross residual tumor) whenever it is possible. Nevertheless, due to the high rate of recurrent tumors (40–60%) and the low median survival rate of 16–40 months post-diagnosis, newer and more effective methods of treatment need to be developed [14,15]. The pathogenesis and genetic alterations of CSs are still poorly understood, and different theories have been proposed to explain their biphasic nature [16,17]. In multiple studies, the clinically aggressive behavior of CS has been attributed to the epithelial component of the tumor and, as a result, CS has undergone a recent declassification to metaplastic carcinomas [18,19]. Contrary to other gynecological conditions that require, in selected cases, fertility sparing treatment [20,21,22,23,24], patients affected by uterine CS are not currently considered for this approach and radical surgery is the cornerstone of treatment.

Two separate and mutually exclusive models have been developed to explain the development of tumors. The clonal evolution model postulates that all cells within a tumor contribute in varying degrees to the maintenance of the tumor. In this model, a number of genetic and epigenetic changes occur over time, with the result that the most aggressive cancer cells are ultimately responsible for driving tumor progression. Furthermore, through a series of genetic mutations, any cancer cell within the tumor can become invasive, lead to the development of metastases, and contribute to the resistance of therapies and ultimately to the recurrence of disease. The “cancer stem cell” model proposes that these cells are ultimately responsible for tumor initiation, progression and recurrence. It is thought that through self-renewal and differentiation, cancer stem cells are responsible for the production of the various tumor cell types and contribute to tumor heterogeneity. Furthermore, according to this hypothesis, tumor metastases and resistance to therapies arise directly from cancer stem cells [25]. Cancer-initiating stem cells have been isolated in leukaemia and in solid tumors such as breast, lung and gynecologycal malignancies [26]. Cancer stem cells have a role in tumor progression, angiogenesis and metastasis. The biological characteristics of these cells are similar to those of normal stem cells. They have self-renewal ability and indefinite proliferation, both associated with tumor progression [27]. These cells are insensitive to chemotherapy treatments, showing a clonal progression and facilitating recurrence. Tumor microenvironment, such as immune cell infiltrates, plays a key role in neoplastic progression. The immune responses observed in many cancers are attracted by tumor-associated antigens and, as suggested by recent research, by neoantigens-immunogenic antigens encoded for by non-synonymous mutations. The promising efficacy of immune checkpoint inhibitors in the same solid tumors has led researchers to study and develop new agents targeting these immune escape mechanisms. Some recent studies, aimed at improving the management of metastatic breast cancer, showed encouraging results and suggest at least some clinical activities. Due to the abovementioned reasons, there has been an increased interest in literature regarding new therapeutic interventions, such as targeting cellular pathways that drive epithelial cell proliferation, representing a potential benefit in the treatment of these biologically aggressive tumors. The objective of this systematic review was to bring together all the all latest new target therapies for the treatment of CS.

## 2. Results

The search identified 30 citations in total. After the initial evaluation, 23 of these were potentially relevant and were in accordance with the inclusion criteria, hence they were subsequently analyzed (Table 1). No randomized controlled trials (RCTs) were found. We divided all included studies into three different issues: “Human Epidermal Growth Factor family of receptors (ErbB family)”, “Human Epidermal Growth Factor Receptor 2 (HER2)”, and “Epithelial cell adhesion molecule-1 (EpCAM)”.

### 2.1. Genetic Landscape

The development of novel, effective therapies remain desperately necessary. In the last few years, multiple comprehensive genetic studies have reported the mutational landscape of CS, providing an opportunity for the identification of multiple deranged pathways as potential novel targets for the treatment of this highly lethal tumor. Zhao et al. [28] reported on the genetic landscape of uterine and ovarian CS. Further to mutations in cancer genes already observed in uterine and ovarian CS such as *TP53*, *PIK3CA*, *PPP2R1A*, *KRAS*, *PTEN*, *CHD4*, and *BCOR*, this study identified an excess of mutations in genes encoding histone H2A and H2B, demonstrating a stable transgenic expression of H2A and H2B in a uterine serous carcinoma cell line. However, the mutant’s histones were not wild-type, and they increased the expression of epithelial-mesenchymal markers in addition to the tumors’ migratory and invasive properties, indicative of a role in sarcomatous transformation [29]. Multiple groups investigated the HER2 expression levels in uterine and ovarian CS [28,30,31,32,33,34,35,36,37,38,39,40,41,42,43,44]. Raspollini et al. [44] identified HER2 overexpression in seven of 24 CS cases (29.2%), tested by immunohistochemistry. Sawada et al. [45] and Amant et al. [35] also identified higher HER2 overexpression in CS, i.e., nine of 16 cases (56%), and three of seven cases (43%), respectively, when considering both 2+ and 3+ positivity by immunohistochemistry [32,36]. Finally, Livasy et al. [34] reported that up to 25% of CS (14/55 cases) may overexpress HER2 at 3+ levels by immunohistochemistry. Another study reported that surface expression of EpCAM was observed in 80.0% (four out of five) of the CS cell lines tested by flow cytometry [37].

### 2.2. Human Epidermal Growth Factor Family of Receptors (ErbB Family)

The carcinomatous component drives a biphasic growth of the tumor, thereby leading to speculation that therapeutic interventions which target the proliferation of epithelial cells may represent a potential benefit against these biologically aggressive tumors [38]. The ErbB family of receptors incorporates four structurally related members: HER1 (ErbB1, also known as EGFR), HER2 (ErbB2), HER3 (ErbB3), and HER4 (ErbB4). The ErbB family members are receptor tyrosine kinases (RTKs), analogously structured, consisting of an extracellular ligand-binding domain, a single hydrophobic transmembrane region, and an intracellular segment containing a conserved tyrosine kinase domain [39].

The concept of “cancer stem cells” is constantly evolving and remains controversial. Recent scientific papers have shown that cancer stem cells and cancer-initiating cells have similar properties and act uniquely in the cancerogenetic process. It is proved that in a cancer stem cell model, there is a hierarchical organization with a population of self-renewing cells at the basis of cancerogenesis [40]. Following this model, cancer stem cells are considered to be unique subsets of clones with features such as chemotherapy resistance, and are considered to be initiators of the metastatic process. Evidence exists in osteosarcoma that patients may present with distant metastases decades after the completion of their first treatment, potentially further highlighting the tumorigenic characteristics of cancer stem cells, although this currently remains speculative [41]. Recently, it has been shown that a stem cell transcription factor (Sox2) supports cancer stem cells through the inhibition of the Hippo pathway [42]. Furthermore the stem cell niche, which is part of the surrounding microenvironment, regulated the self-renewal process of normal stem cells. This microenvironment influences cancer stem cell function and survival and in some cancers these niches may even overlap with the normal stem cell niche [43]. In addition, genomic instability, dormancy and potential new resistant mechanisms can be modified by the stem cell niche [43].

### 2.3. HER2

**Figure 1:**
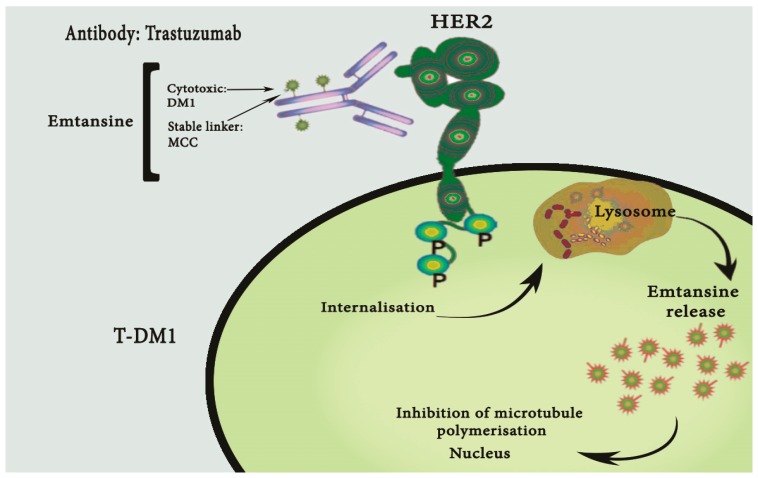
Mechanism of action of Trastuzumab-Emtasine (T-DM1).

It is known that HER2 plays a crucial part in the growth, survival and differentiation of cells, both in normal and tumor cells, through the enhancement of kinase-mediated activation of downstream signaling pathways. These pathways involve mitogen-activated protein kinase and phosphatidylinositide 3-kinase [36,37]. The *HER2/neu* oncogene has been reported as overexpressed and/or amplified in various human tumor types, including CS of the female genital tract [44,45,46,47,48,49]. Cancers that overexpress HER2 have been linked with worse prognosis when compared with matched *HER2* non-amplified ones, including a higher mortality rate in early stages of the disease, reduced relapse time, and a higher incidence of metastases [50,51]. Trastuzumab emtansine (T-DM1, Kadcyla™, Genentech, South San Francisco, CA, USA) is a new HER2-targeting immunotoxin which was developed through a combination of Trastuzumab (T) (Herceptin, Genentech, South San Francisco, CA, USA) with maytansinoid cytotoxin (DM1), which significantly inhibits the polarization of microtubules (Figure 1) [52]. For this reason, T-DM1 binds the extracellular sub-domain IV of the HER2 receptor after internalization. At this point, the release of DM1 can cause the cell cycle arrest and consequent apoptosis [53]. Since 2012, HER2 has been identified as a possible therapeutic target in CS and opened new scenarios for its possible treatment [54]. The introduction of T in the treatment of HER-2-positive metastatic breast cancer patients favorably changed the natural history of this disease. First-line treatment with T and taxanes demonstrated a significant improvement in the overall survival and progression-free survival rates compared with single drug treatment alone. Numerous clinical trials have shown how the subgroup of patients with HER-2-positive metastatic breast cancer are more likely to face a disease progression. In cases of patients previously treated with trastuzumab and taxanes, TDM-1 shows better results and less toxicity than lapatinib plus capecitabine [55]. Although breast, ovarian, gastric, uterine and other solid cancers have notable distinctions, there may exist parallel pathways that can be targeted: for example, CLEOPATRA (NCT00567190), a Phase III randomized clinical trial studying pertuzumab in women with HER2-amplified metastatic breast cancer, changed the clinical practice since 2014. Its counterpart, the Phase III randomized PENELOPE trial (NCT01684878), was activated following promising Phase II data and studied pertuzumab in an enriched ovarian cancer patient population with low HER3 mRNA [56]. The addition of the HER2-directed antibody trastuzumab to chemotherapy also increased the overall survival rate of patients with metastatic HER2-positive esophago-gastric cancer, although targeting HER2 still remains challenging due to the complex biology of this receptor in gastric and esophageal cancers. With standardized HER2 testing in gastro-esophageal cancer, the ongoing trials are testing newer agents and the combination of anti-HER2 antibodies with immunotherapy [57]. In any case, clonal heterogeneity and the emergence of resistance will be the most important challenges that we will have to counteract. After 2015, the efficacy of T-DM1 was evaluated against primary HER2-positive and HER2-negative CS cell lines in both in vitro and in vivo studies [2]. According to this study, the overexpression of HER2 protein and gene amplification were observed in 25% (2/8 cases) of the primary CS cell lines. T-DM1 and T were similarly effective in inducing strong antibody-dependent cell-mediated cytotoxicity (ADCC) against CS overexpressing HER2 at 3+ levels. In contrast, T-DM1 was significantly more effective than T in the inhibition of cell proliferation (*p* < 0.0001) and in the induction of G2/M phase cell cycle arrest in the HER2 expressing cell lines (shift of G2/M: mean ± Standard Error of the Mean (SEM) from 14.87 ± 1.23% to 66.57 ± 4.56%, *p* < 0.0001). Similarly, T-DM1 was highly active in the reduction of tumor formation in vivo in control xenografts that overexpressed HER2 (*p* = 0.0001 and *p* < 0.0001 compared to T and vehicle, respectively), with a highly longer survival time in comparison with T and vehicle mice (*p* = 0.008 and *p* = 0.0001, respectively) [2]. T-DM1 could therefore indicate a new treatment option for the subset of HER2-positive CS patients with disease refractory to chemotherapy. In another well-designed experiment [58], HER2/neu was found amplified in 28.5% of CS cell lines. These cells lines, named SARARK6 and SARARK9, were identified as being more sensitive to neratinib with respect to the other non-HER2/neu amplified cell lines (mean ± SEM IC50: 0.014 ± 0.004 vs. 0.164 ± 0.019 micromole (μM), *p* = 0.0003). In particular, neratinib was able to arrest the activation of S6 and autophosphorylation of HER2/neu, as well as increase the G0/G1 phase of the cell cycle. Finally, it was found to inhibit tumor growth (*p* = 0.012) and to prolong survival in mice harboring HER2-amplified carcinosarcoma xenografts (*p* = 0.0039).

### 2.4. Epithelial Cell Adhesion Molecule-1 (EpCAM)

When epithelial cell adhesion molecule-1 (EpCAM), also named TROP-1 or TACSTD1, is overexpressed, this could be considered a prognostic biomarker for several carcinomas and CS [59,60]. EpCAM was first described in 1979 as an antigen, which induced the production of specific antibodies after the immunization of mice with human colorectal carcinoma cells and the subsequent fusion of splenocytes with myeloma cells to create hybridomas [61]. The mid-1990s brought about large clinical phase studies utilizing the monoclonal anti-EpCAM antibody Edrecolomab [62,63], which resulted in Food and Drug Administration (FDA) approval and market introduction of Panorex for the treatment of metastasized colon cancer. In parallel to this development, others focused on the molecular and functional aspects of EpCAM to reveal its role as a homophilic cell adhesion molecule and a correlation to proliferation [64,65]. It must be noted that Panorex was retrieved from the German market in 2000 and the actual benefit of this particular anti-EpCAM antibody was eventually refuted in a large cohort of patients suffering from stage III colon cancer [66]. Besides intense discussions and arguments on reasons for such a clinical failure even after FDA approval [67], it appears advisable to heed the following important issues when considering EpCAM as a target for therapy: expression levels of EpCAM matter with respect to the potential benefit of EpCAM-specific antibody therapy, thus the effects of anti-EpCAM antibody on EpCAM signaling must be paid attention to and the inhibition of EpCAM signaling with specific inhibitors of regulated intramembrane proteolysis is a novel option to be considered [68].

In the great majority of cancer entities, EpCAM overexpression strongly correlates with worse overall survival rate and poor prognosis [69], and distinguishes patients at high risk for recurrence [70]. However, in some entities such as pancreatic and gastrointestinal cancers, EpCAM overexpression correlates with better prognosis [55]. EpCAM is 39–42 kDA protein and consist of three domains: a short 26 amino acid cytoplasmic domain, a transmembrane domain, and an extracellular domain with two epidermal growth factor-like repeats. This protein is expressed at low levels on several epithelia and promotes cell adhesion. It is significantly expressed in epithelial tissues in the female genital tract and in the basolateral and intercellular surface of simple, pseudo-stratified, and transitional epithelia [56]. It is widely accepted that EpCAM have paramount importance for cell differentiation, proliferation, signaling and migration. In addition, EpCAM could be considered an interesting target for immunotherapy, since it is highly expressed on the cell surface of multiple human carcinomas [65]. In this regard, Solitomab (MT110, AMG 110), an EpCAM/CD3-bispecific single-chain antibody, is able to bind polyclonal CD8+ and CD4+ T cells and activate them, addressing against EpCAM-expressing tumor cells [71].

**Figure 2:**
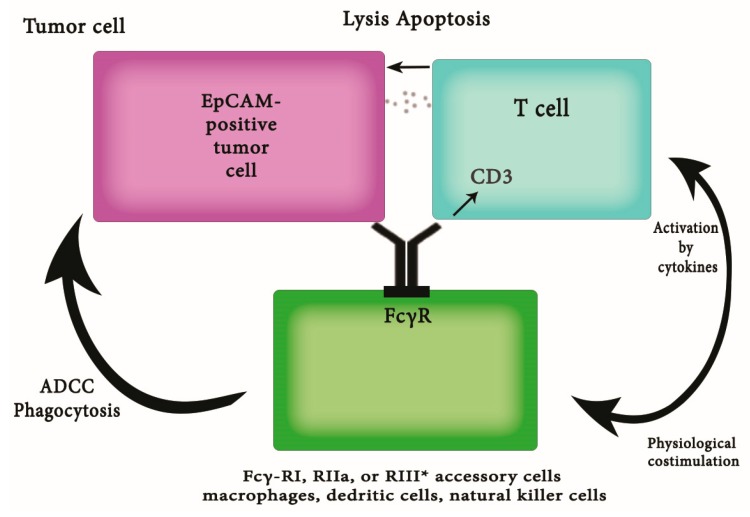
Mechanism of action of Catumaxomab (Removab).

During the last few decades, several efforts were made to generate therapeutic antibodies. The first clinical studies enrolling patients treated with Edrecolomab revealed a reduced tumor recurrence and death rate, whereas these effects could not be reproduced in larger clinical trials [72]. This failure of clinical response might be due to the short serum half-life of the murine antibody and to a lack of randomization of patients according to their actual EpCAM status on tumor cells. Clearly, it is expected that only patients with high-level EpCAM expression on tumor cells would benefit from antibody treatment and therefore randomization appears mandatory. As a matter of fact, knowledge on the actual function of a target will help to develop more effective therapeutics with lower side-effects, as has been beautifully shown for the case of Her and T. By the time EpCAM-specific antibodies were developed, knowledge on EpCAM’s capacities to signal and induce proliferation was not available. Accordingly, differences in the influence of EpCAM-specific antibodies on the proliferation of EpCAM-positive cells have recently been demonstrated [73] and must be taken into consideration for future developments. In 2009, the first antibody targeting EpCAM, called Catumaxomab (Removab), obtained approval for the European market. This trifunctional antibody has the ability to bind EpCAM-expressing cancer cells as well as cytotoxic T-cells via the CD3 receptor. The Fcγ receptor allows the molecule to further bind and activate accessory cells such as macrophages, Natural Killer (NK) cells, and Dendritic Cells (DCs) (Figure 2) [74]. Clinical trials revealed humoral responses against this antibody after treatment. This might be due to the chimeric structure consisting of mouse IgG2a and rat IgG2b. Surprisingly, this kind of response against Catumaxomab correlated with the clinical outcome in a positive manner and treatment of patients with malignant ascites with Catumaxomab caused a prolonged overall survival rate [75]. So far, all clinical trials reported herein did not specifically deal with the eradication of Tumor Infiltrating Cells (TICs) in particular, but rather with the treatment of EpCAM-positive cancers. Recently, the bispecific antibody MT110 was tested for its ability to target TICs derived from colorectal cancers. This antibody has binding affinities for EpCAM and CD3, which allow it to initiate the formation of a cytolytic synapse between T-cells and TICs. A combination of the antibody and PBMCs (Peripheral Blood Mononuclear Cells) led to decreased or absent colony formation in soft agar assays. Additionally, treatment with MT110 prevented tumor formation in a xenograft model, where mice were inoculated with TICs [76]. Shigdar et al. generated a specific nuclease resistant aptamer targeting EpCAM, known as EpDT3 [77]. This molecule consisting of only 19 nucleotides offers a similar moderate binding affinity as, for instance, Catumaxomab or Adecatumumab antibodies. Importantly, EpDT3 is internalized into the cell via endocytosis after binding to EpCAM on the surface. Hence, this mechanism can be used to channel diverse substances such as chemotherapeutic drugs, toxins and therapeutic radioisotopes. The high accessibility due to its small molecular size is considered to be an advantage compared with antibodies. For the case of EpCAM it could however be a disadvantage, since EpCAM-expressing healthy tissue might be targeted as well [77]. Based on the novel understanding of the functions of EpCAM, another interesting approach relies on the interference with the signaling cascade of EpCAM. The knowledge of proteases involved in the activating proteolytic cleavage of EpCAM allows for the systematic testing of combinations of inhibitors of these proteases, i.e., tumor necrosis factor-alpha converting enzyme (TACE) and presenilin 2, therapeutic antibodies, and conventional treatments involving chemotherapeutics. Even more interesting, the inhibition of epithelial cell intra-cellular domain (EpICD) bind with four and a half LIM domain 2 (FHL2) interaction by small molecules generated upon structure-based rational design and bioinformatics is a promising and highly innovative strategy to specifically target EpCAM and its signaling. In vitro experiments support the inhibitory effects of TACE and gamma-secretase inhibitors on EpCAM-dependent proliferation and target gene induction. Likewise, the knockdown of FHL2 resulted in an inhibition of EpCAM-specific effects [73]. Last but not least, the differential cleavage and localization of EpICD in normal and malignant colon epithelium represents a solid in vivo rationale for a therapeutic intervention at the level of EpCAM cleavage and signaling. Ferrari et al. [33] showed EpCAM surface expression in four out of five CS cell lines studied by flow cytometry; interestingly, EpCAM-expressing cell lines showed resistance to NK or T-cell-mediated killing after exposure to peripheral blood lymphocytes (PBL) in 4-h chromium-release assays (mean killing ± SEM = 1.1 ± 1.6%, range 0–5.3% after incubation of EpCAM-positive cell lines with control BiTE^®^). Conversely, EpCAM-positive CS cells became highly sensitive to T-cell-cytotoxicity (mean killing ± SEM of 19.7 ± 6.3%; range 10.0–32.0%; *p* < 0.0001) after incubation with solitomab. In addition, ex vivo incubation of autologous tumor-associated lymphocytes with EpCAM-expressing malignant cells in pleural effusion with solitomab resulted in a high increase of T-cell activation markers (i.e., CD25 and human leukocyte antigens D Related (HLA-DR), CD4+ and CD8+ T cell proliferation, as well as a reduction of viable CS cells in the exudate (*p* < 0.001). For these reasons, solitomab could represent an effective treatment for patients with recurrent, metastatic, and/or chemoresistant CS overexpressing EpCAM [33,78].

## 3. Discussion

Gynecological CSs are very infrequent and represent only 2–5% of uterine cancers. Despite surgical cytoreduction and subsequent chemotherapy being considered the best primary treatment for uterine CS, the overall five-year survival rate is 30 ± 9% for all stages and recurrence is very common (50–80%) [5,6,38]. The characteristics of uterine CS are very different from other types of gynecological cancer, such as ovarian [79,80,81,82,83,84,85], endometrial [86,87], cervical [88,89] and vulvar [90,91] cancers. The atypical pathogenesis and poor prognosis of uterine CS, despite the aggressive conventional treatments, have pushed researchers to understand the molecular basis of CS, with the aim to individuate new and effective methods of target treatment. Historically, Cohnheim elaborated the theory that tumors may arise from stem cells which remain after embryonic development [92]. So, it was deducted that self-renewing cancer stem cells drive cancerogenesis, and that these cells are able to generate differentiated cell populations creating the tumor cell mass [93]. In this regard, the pioneer work by Gorai et al. [94] on the EMTOKA cell line and its clones clearly addresses the combination tumor hypothesis; according to this theory, a precursor (stem) cell gives rise both to epithelial and to mesenchymal components during the histogenesis of uterine carcinosarcoma, the epithelial component of which appears to be dominant, suggesting that the established cell lines derived from a common stem cell. Cancer stem cells were first isolated from leukemia samples in 1997 [26] and, later, from breast and endometrial cancer [95,96] and from many other solid tumors including several types of sarcoma, as well as from uterine myometrium [97]. More recently, it has been showed that CD133+ cells purified from endometrioid adenocarcinomas are resistant to cisplatin-induced and paclitaxel-induced cytotoxicity and express a peculiar gene signature consisting of high levels of matrix metalloproteases, interleukin-8, CD44, and CXCR4. Interestingly, when these cells are serially transplanted into NOD/SCID mice, they have been shown to recapitulate the phenotype of the original tumor [96]. Although data are still not so robust as to draw firm conclusions, it was recently shown that side populations of myometrial cells share similar stem potentials with respect to cancer stem cells [97]. In particular, they can also generate functional human myometrial tissues efficiently when transplanted into the uteri of severely immunodeficient mice and, additionally, are able to differentiate into osteocytes and adipocytes in vitro under the appropriate differentiation-inducing conditions.

Cancer stem cells possess strong tumorigenicity and metastacity, as well as radio and chemo resistance. It is clear that these cells play a key role in neoplastic progression [98,99].

Cancer stem cells also comprise a small population of cells within a tumor, known as “tumorigenic cells”. Normal stem cells and cancer stem cells have similar cell surface markers [100], self-renewal ability, and develop into different progeny [101]. However, the proportion of tumorigenic cells is not always equal even among tumors of the same histotype [102].

Emerging theories indicate that cancer stem cells are a cell “status” but not a fixed “category” of cells. Cancer stem cells are not an immutable, frozen cell population. Cancer stem cells and non-cancer stem cells coexist and are linked in a dynamic equilibrium [103]. The microenvironment is a determining factor in this signal progression; for example, myofibroblast-secreted factors restore cancer stem cell phenotypes in differentiated colon cancer cells in vitro and in vivo [104].

The role of vascular endothelial growth factor (VEGF) in tumor angiogenesis is proven by a series of experiments, using monoclonal antibodies targeting the vascular endothelial growth factor VEGF. Angiogenesis in experimental tumors could be restricted by anti-VEGF treatment, and this in turn restricted the solid tumor growth [105]. The humanized VEGF antibody, known as Avastin^TM^ or bevacizumab, has been approved by the FDA for treating metastatic carcinoma of the colon or rectum [106] and recurrent or metastatic non-squamous non-small cell lung cancer [107]. Recently, Avastin^TM^ also received accelerated FDA approval for treatment of metastatic HER2-negative breast cancer. The years of research to uncover key angiogenic growth factors and their receptors has driven considerable interest in the broader area of endothelial cell biology.

## 4. Materials and Methods

### 4.1. Data Sources

We searched MEDLINE (PubMed), EMBASE, Cochrane Central Register of Controlled Trials, IBECS, BIOSIS, Web of Science, SCOPUS, congress abstracts, and grey literature (Google Scholar; British Library, London, UK) from January 1952 to August 2016. We performed a systematic review using the terms “uterine carcinosarcoma”, “uterine Malignant Mixed Müllerian Tumor”, “target therapy”, “angiogenesis therapy”, “cancer stem cell therapy”, “prognostic biomarker”, and “novel antibody-drug”. A second search was performed, screening the reference lists of all the primary available studies, in order to identify additional citations that could be relevant.

### 4.2. Screening of Abstracts for Eligibility

Abstracts/titles identified from the search were screened by three investigators (Gaetano Valenti, Fabrizio Sapia and Giuseppe Sarpietro.). Disagreements regarding the inclusion or exclusion of studies were initially solved by consensus. When this was impossible, they were arbitrarily resolved by a fourth reviewer (Salvatore Lopez).

### 4.3. Study Selection and Eligibility Criteria

Explicit criteria were chosen to be used in the selection of the literature: (1) original articles, (2) clinical trials conducted on culture, human/animal species and (3) English language.

## 5. Conclusions

Based on our results, the differential expression and accessibility of EpCAM on metastatic/chemotherapy-resistant CS cells in comparison with normal tissues and HER2 open up new possibilities in the field of targeted therapy [12]. However, much work is still required to improve the survival rate and quality of life of women with CS. Until now, the addition of target therapies to conventional chemotherapy seemed to be the most promising option, significantly contributing to an improvement in response and survival rates. It would also be interesting to extend larger studies on first-line treatment to improve the outcome in these patients prior to recurrence. In addition, a larger screening project is necessary and should be encouraged in order to reduce the incidence rate of this disease worldwide. Last but not least, the economic impact of the use of target therapies comes with an indisputably high cost, particularly if we consider that these patients have a poor prognosis and the median survival at five years is around 5% for metastatic disease. Thus, how could we optimize costs in favor of a better outcome with an acceptable quality of life for our patients? This could be also an important objective in the future.

## Figures and Tables

**Table 1:** Flowchart for the selection of the studies.

**30 Potentially Citations Identified and Screened for Retrieval**	
Primary survey: excluded	three studies about other cancer types
	two studies about benign disease or healthy women
**25 potentially eligible studies retrieved for more detailed evaluation**	
Secondary survey: excluded	two studies available only in non-English language
23 eligible studies identified	

## Abbreviations

ADCC	Antibody-Dependent Cell-Mediated Cytotoxicity
AT	Adjuvant Therapy
CS	Carcinosarcoma
CSCs	Cancer Stem Cells
DM1	Maytansinoid Cytotoxin
EMA	Epithelial Membrane Antigen
ErbB family	Human Epidermal Growth Factor Family of Receptors
FDA	Food and Drug Administration
FIGO	International Federation of Gynecology and Obstetrics
HER2	Human Epidermal Growth Factor Receptor 2
MMT	Malignant Mixed Müllerian Tumor
CD10	Cluster of Differentiation 10
EpCAM	Epithelial Cell Adhesion Molecule-1
SEM	Standard Error of the Mean
T	Trastuzumab
T-DM1	Trastuzumab Emtansine
VEGF	Vascular Endothelial Growth Factor
μM	Micromole
NK	Natural Killer
DCs	Dendritic Cells
TACE	Tumor Necrosis Factor-Alpha Converting Enzyme
EpICD	Epithelial Cell Intra-Cellular Domain
FHL2	Four and a Half LIM Domain 2
HLA-DR	Human Leukocyte Antigens D Related
TICs	Tumor Infiltrating Cells
